# Dibenzo[*b*,*f*][1,4]thia­zepin-11-yl-diethyl-amine

**DOI:** 10.1107/S1600536812042328

**Published:** 2012-10-13

**Authors:** Maria Altamura, Antonio Guidi, Loic Jierry, Paola Paoli, Patrizia Rossi

**Affiliations:** a Chemistry Department, Menarini Ricerche S.p.A., Via dei Sette Santi 3, I-50131 Firenze, Italy; b ICS, Université de Strasbourg, Strasbourg, France; c Dipartimento Energetica "Sergio Stecco", University of Firenze, Via S. Marta 3, I-50139 Firenze, Italy

## Abstract

In the title compound, C_17_H_18_N_2_S, the thia­zepine ring adopts a boat conformation and the dihedral angle between the benzene rings is 75.92 (5)°, resulting in a butterfly-like conformation. In the crystal, mol­ecules are connected *via* weak C_aromatic_—H⋯N contacts involving the imine N atom as acceptor and through a quite short C—H⋯π inter­action. The resulting mol­ecular chains propagate along the *c-*axis direction.

## Related literature

For ‘privileged structures’, that is ‘structures able to provide high affinity ligands for more than one type of receptor’, see: Evans *et al.* (1988 ▶); Patchett & Nargund (2000 ▶); Fedi *et al.* (2008 ▶). For the clinical use of dibenzothia­zepine derivatives, see: Ganesh *et al.* (2011 ▶); Pettersson *et al.* (2009 ▶); Riedel *et al.* (2007 ▶); Warawa *et al.* (2001 ▶). For structure–property relationships in (6,7,6)-tricyclic ring systems, see: Ravikumar & Sridhar (2005 ▶); Altamura *et al.* (2008 ▶, 2009 ▶, 2011 ▶). For geometrical data and descriptors, see: Duax *et al.* (1976 ▶); Bertolasi *et al.* (1982 ▶); Allen *et al.* (1987 ▶).
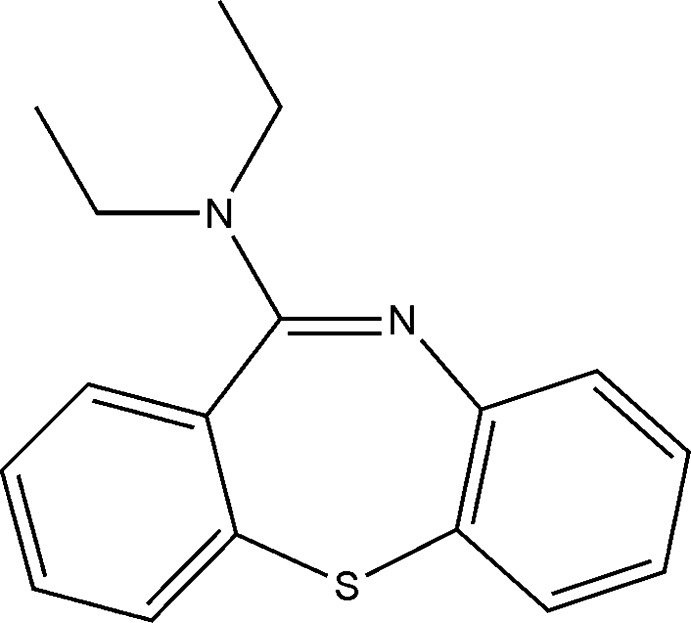


## Experimental

### 

#### Crystal data

C_17_H_18_N_2_S
*M* *_r_* = 282.40
Monoclinic, 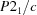

*a* = 12.0137 (2) Å
*b* = 8.2257 (1) Å
*c* = 15.0513 (2) Å
β = 102.952 (1)°
*V* = 1449.54 (4) Å^3^
*Z* = 4
Cu *K*α radiation
μ = 1.89 mm^−1^
*T* = 150 K
0.20 × 0.18 × 0.03 mm

#### Data collection

Oxford Diffraction XcaliburPX diffractometer
Absorption correction: multi-scan (*CrysAlis RED*; Oxford Diffraction, 2006 ▶) *T*_min_ = 0.722, *T*_max_ = 0.945
6173 measured reflections
2364 independent reflections
1837 reflections with *I* > 2σ(*I*)
*R*_int_ = 0.019

#### Refinement

*R*[*F* ^2^ > 2σ(*F* ^2^)] = 0.032
*wR*(*F* ^2^) = 0.086
*S* = 1.05
2364 reflections
181 parameters
H-atom parameters constrained
Δρ_max_ = 0.23 e Å^−3^
Δρ_min_ = −0.26 e Å^−3^

### 

Data collection: *CrysAlis CCD* (Oxford Diffraction, 2006 ▶); cell refinement: *CrysAlis CCD*; data reduction: *CrysAlis RED* (Oxford Diffraction, 2006 ▶); program(s) used to solve structure: *SIR92* (Altomare *et al.*, 1999 ▶); program(s) used to refine structure: *SHELXL97* (Sheldrick, 2008 ▶); molecular graphics: *ORTEP-3* (Farrugia, 1997 ▶); software used to prepare material for publication: *PARST* (Nardelli, 1995 ▶).

## Supplementary Material

Crystal structure: contains datablock(s) I, New_Global_Publ_Block. DOI: 10.1107/S1600536812042328/nc2296sup1.cif

Structure factors: contains datablock(s) I. DOI: 10.1107/S1600536812042328/nc2296Isup2.hkl

Additional supplementary materials:  crystallographic information; 3D view; checkCIF report

## Figures and Tables

**Table 1:** Hydrogen-bond geometry (Å, °) *Cg* is the centroid of the C8–C12 ring.

*D*—H⋯*A*	*D*—H	H⋯*A*	*D*⋯*A*	*D*—H⋯*A*
C5—H5⋯N1^i^	0.95	2.70	3.576 (2)	154
C4—H4⋯*Cg* ^i^	0.95	2.81	3.5759 (18)	139

Symmetry code: (i) 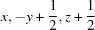.

**Fig. 1.**
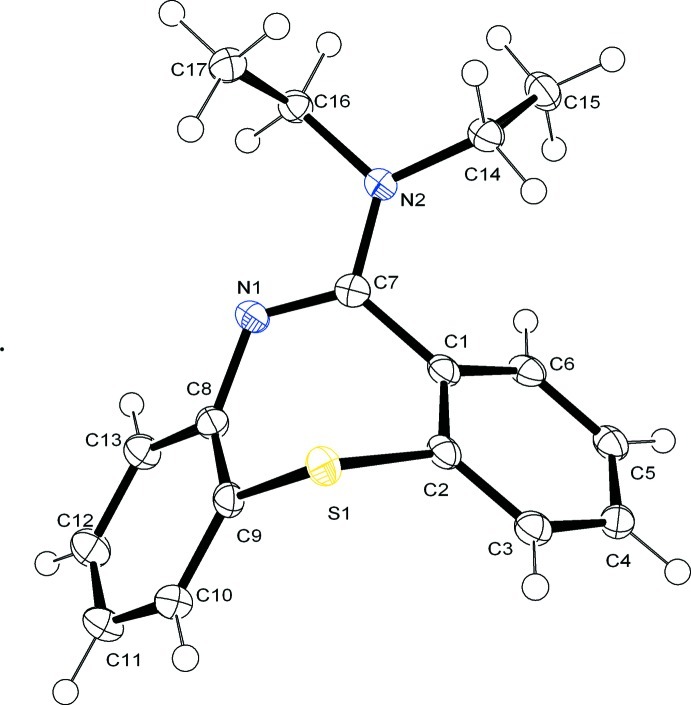
Crystal structure of the title compound with labelling and displacement ellipsoids drawn at the 50% probability level.

**Fig. 2.**
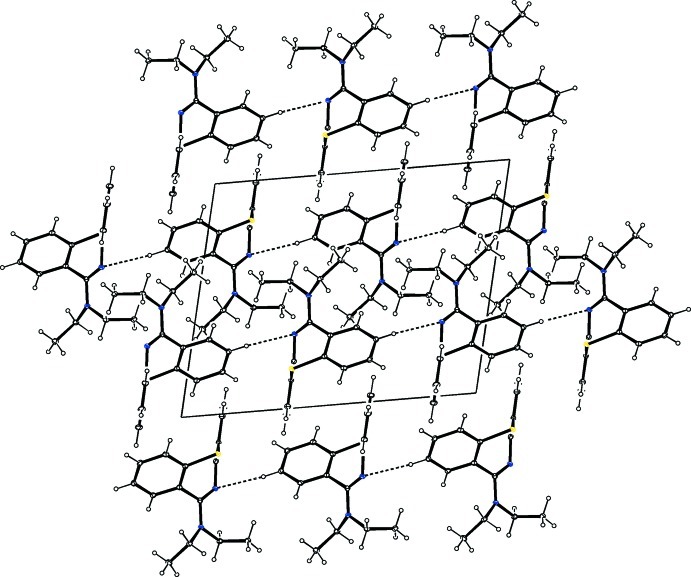
Crystal structure of the title compound with view along the b-axis. Intermolecular H-bonding interactions are shown as dashed lines.

## supplementary crystallographic information

### Comment

Many antidepressant drugs have a tricyclic structure with two aromatic rings fused to a central seven membered ring, on which one or more heteroatoms can be present. In this context, dibenzothiazepines are a class of heterocyclic scaffolds containing nitrogen and sulfur which belong to the class of the so-called "privileged structures", *i.e.* structures "able to provide high affinity ligands for more than one type of receptor" (Evans *et al.* 1988; Patchett & Nargund 2000; Fedi *et al.* 2008). In fact the dibenzothiazepine skeleton has a broad spectrum of medical applications; its derivatives are used to treat schizophrenia and also find applications as neuroleptics, antidepressants, antihistaminic, just to name a few (Ganesh *et al.* 2011; Pettersson *et al.* 2009; Riedel *et al.* 2007; Warawa *et al.* 2001). Within our programme research concerning the structural elucidation of tricyclic molecules in order to gain further insights into structure–property relationships (Altamura *et al.*, 2008; Altamura *et al.*, 2009; Altamura *et al.*, 2011) we investigated the crystal and molecular structure of the title compound. The overall shape of the tricyclic skeleton is controlled both by the conformation of the central seven-membered ring and the relative arrangement of the aromatic rings bound to it. The central thiazepine ring adopts the usual boat conformation, with C1, C2, C8 and C9 as the basal plane, the S atom as the bow and the N1—C7 bond as the stern. The deviation from a pure boat conformation is quite small as can be seen from the asymmetry index ΔC_s_ which is 3.97°; the bow angle is 51.04 (8)° and the stern angle is 41.89 (8)°(Duax *et al.* 1976; Bertolasi *et al.* 1982; Ravikumar & Sridhar, 2005). Finally, the dihedral angle between the benzene rings is 104.08 (5)°. As a consequence the dibenzothiazepine tricyclic skeleton assumes an overall butterfly-like shape (Fig. 1). The N2—C7 bond is shorter than an usual N—C single bond [1.368 (2) Å compared to 1.416 Å (Allen *et al.* 1987)] and the sum of the bond angles about N2 is 358°; consequently, N2 has a partial *sp*^2^ character. Molecular chains, which propagate along the *c* axis, are formed through intermolecular interactions (Fig. 2): a weak C—H_aromatic_···N contact which involves the imine nitrogen atom as acceptor and a quite short C—H···π interaction (Table 1).

### Experimental

The synthesis of the title compound started from the commercially available tricyclic lactam (dibenzo[*b,f*][1,4]thiazepin-11(10*H*)-one) (0.89 g, 3.91 mmol), that was dissolved into 10 ml of phosphorus oxychloride and refluxed for two hours under nitrogen atmosphere. After removal of excess POCl_3_ under vacuum, the corresponding iminochloride (11-chlorodibenzo[*b,f*][1,4]thiazepine) was obtained as a yellow oil, that was dissolved in 20 ml of anhydrous toluene. An excess of diethylamine (20 ml, 191 mmol) was added and the mixture refluxed until complete conversion (monitored by HPLC; about 6 h were needed for complete conversion). After removal of the solvent *in vacuo* and purification by flash chromatography (eluent: gradient CH_2_Cl_2_/cyclohexane from 50:50 to 100% CH_2_Cl_2_), dibenzo[b,f][1,4]thiazepin-11-yl-diethyl-amine was obtained as a yellow oil (0.66 g, 60% yield), which became a white solid on standing at room temperature for several days. Crystals suitable for single-crystal X-ray diffraction analysis were obtained by slow evaporation of a water/DMSO solution of dibenzo[b,f][1,4]thiazepin-11-yl-diethyl-amine.

### Refinement

All the H atoms were positioned with idealized geometry using a riding model and refined with *U*_iso_(H) 1.2 times *U*_eq_(C) (1.5 for methyl H atoms).

### Crystal data

**Table:**

C_17_H_18_N_2_S	*F*(000) = 600
*M**_r_* = 282.40	*D*_x_ = 1.294 Mg m^−^^3^
Monoclinic, *P*2_1_/*c*	Cu *K*α radiation, λ = 1.5418 Å
*a* = 12.0137 (2) Å	Cell parameters from 3716 reflections
*b* = 8.2257 (1) Å	θ = 3.8–64.6°
*c* = 15.0513 (2) Å	µ = 1.89 mm^−^^1^
β = 102.952 (1)°	*T* = 150 K
*V* = 1449.54 (4) Å^3^	Platelet, colourless
*Z* = 4	0.20 × 0.18 × 0.03 mm

### Data collection

**Table:**

Oxford Diffraction XcaliburPX diffractometer	2364 independent reflections
Radiation source: Enhance (Cu) X-ray Source	1837 reflections with *I* > 2σ(*I*)
Graphite monochromator	*R*_int_ = 0.019
Detector resolution: 8.1241 pixels mm^-1^	θ_max_ = 64.7°, θ_min_ = 3.8°
ω scans	*h* = −13→11
Absorption correction: multi-scan (*CrysAlis RED*; Oxford Diffraction, 2006)	*k* = −9→9
*T*_min_ = 0.722, *T*_max_ = 0.945	*l* = −16→17
6173 measured reflections	

### Refinement

**Table:**

Refinement on *F*^2^	Primary atom site location: structure-invariant direct methods
Least-squares matrix: full	Secondary atom site location: difference Fourier map
*R*[*F*^2^ > 2σ(*F*^2^)] = 0.032	Hydrogen site location: inferred from neighbouring sites
*wR*(*F*^2^) = 0.086	H-atom parameters constrained
*S* = 1.05	*w* = 1/[σ^2^(*F*_o_^2^) + (0.0564*P*)^2^] where *P* = (*F*_o_^2^ + 2*F*_c_^2^)/3
2364 reflections	(Δ/σ)_max_ < 0.001
181 parameters	Δρ_max_ = 0.23 e Å^−^^3^
0 restraints	Δρ_min_ = −0.26 e Å^−^^3^

### Special details

**Table:**

Geometry. All s.u.'s (except the s.u. in the dihedral angle between two l.s. planes) are estimated using the full covariance matrix. The cell s.u.'s are taken into account individually in the estimation of s.u.'s in distances, angles and torsion angles; correlations between s.u.'s in cell parameters are only used when they are defined by crystal symmetry. An approximate (isotropic) treatment of cell s.u.'s is used for estimating s.u.'s involving l.s. planes.
Refinement. Refinement of *F*^2^ against ALL reflections. The weighted *R*-factor *wR* and goodness of fit *S* are based on *F*^2^, conventional *R*-factors *R* are based on *F*, with *F* set to zero for negative *F*^2^. The threshold expression of *F*^2^ > 2σ(*F*^2^) is used only for calculating *R*-factors(gt) *etc*. and is not relevant to the choice of reflections for refinement. *R*-factors based on *F*^2^ are statistically about twice as large as those based on *F*, and *R*- factors based on ALL data will be even larger.

### Fractional atomic coordinates and isotropic or equivalent isotropic displacement parameters (Å^2^)

**Table:**

	*x*	*y*	*z*	*U*_iso_*/*U*_eq_	
S1	0.83045 (3)	0.13292 (5)	0.85602 (3)	0.01958 (15)	
N1	0.67962 (11)	0.44375 (17)	0.84814 (9)	0.0179 (3)	
N2	0.51660 (11)	0.33602 (17)	0.87760 (9)	0.0181 (3)	
C1	0.69759 (13)	0.2400 (2)	0.97172 (11)	0.0177 (4)	
C2	0.78691 (14)	0.1378 (2)	0.96160 (11)	0.0175 (4)	
C3	0.84541 (14)	0.0447 (2)	1.03477 (11)	0.0198 (4)	
H3	0.9047	−0.0264	1.0268	0.024*	
C4	0.81731 (14)	0.0556 (2)	1.11916 (11)	0.0211 (4)	
H4	0.8564	−0.0089	1.1687	0.025*	
C5	0.73182 (14)	0.1613 (2)	1.13056 (11)	0.0211 (4)	
H5	0.7137	0.1715	1.1886	0.025*	
C6	0.67280 (13)	0.2522 (2)	1.05782 (11)	0.0197 (4)	
H6	0.6143	0.3241	1.0666	0.024*	
C7	0.63322 (13)	0.3418 (2)	0.89419 (11)	0.0169 (4)	
C8	0.79782 (14)	0.4649 (2)	0.85930 (11)	0.0188 (4)	
C9	0.87701 (14)	0.3391 (2)	0.85923 (11)	0.0188 (4)	
C10	0.99058 (14)	0.3727 (2)	0.85997 (11)	0.0237 (4)	
H10	1.0430	0.2861	0.8604	0.028*	
C11	1.02769 (15)	0.5322 (2)	0.86002 (12)	0.0290 (5)	
H11	1.1051	0.5551	0.8596	0.035*	
C12	0.95124 (15)	0.6578 (2)	0.86075 (12)	0.0280 (5)	
H12	0.9767	0.7673	0.8618	0.034*	
C13	0.83815 (14)	0.6251 (2)	0.86002 (11)	0.0218 (4)	
H13	0.7867	0.7128	0.8600	0.026*	
C14	0.45010 (14)	0.2064 (2)	0.90866 (11)	0.0200 (4)	
H14A	0.5032	0.1237	0.9421	0.024*	
H14B	0.4014	0.1529	0.8548	0.024*	
C15	0.37461 (15)	0.2692 (2)	0.97035 (12)	0.0252 (4)	
H15A	0.3324	0.1782	0.9891	0.038*	
H15B	0.3206	0.3493	0.9371	0.038*	
H15C	0.4224	0.3203	1.0245	0.038*	
C16	0.45022 (14)	0.4423 (2)	0.80635 (11)	0.0188 (4)	
H16A	0.4865	0.5510	0.8112	0.023*	
H16B	0.3723	0.4555	0.8170	0.023*	
C17	0.44140 (14)	0.3772 (2)	0.71044 (11)	0.0209 (4)	
H17A	0.3965	0.4527	0.6661	0.031*	
H17B	0.4039	0.2707	0.7046	0.031*	
H17C	0.5181	0.3662	0.6988	0.031*	

### Atomic displacement parameters (Å^2^)

**Table:**

	*U*^11^	*U*^22^	*U*^33^	*U*^12^	*U*^13^	*U*^23^
S1	0.0205 (2)	0.0207 (3)	0.0190 (2)	−0.00074 (18)	0.00747 (17)	−0.00055 (18)
N1	0.0153 (7)	0.0199 (8)	0.0191 (7)	−0.0019 (6)	0.0049 (6)	−0.0011 (6)
N2	0.0153 (7)	0.0197 (8)	0.0196 (8)	−0.0014 (6)	0.0042 (6)	0.0016 (6)
C1	0.0148 (8)	0.0195 (9)	0.0187 (9)	−0.0059 (7)	0.0034 (7)	−0.0009 (7)
C2	0.0163 (9)	0.0188 (9)	0.0177 (9)	−0.0058 (7)	0.0041 (7)	−0.0019 (7)
C3	0.0170 (9)	0.0189 (10)	0.0232 (9)	−0.0023 (7)	0.0041 (7)	−0.0009 (8)
C4	0.0190 (10)	0.0234 (10)	0.0190 (9)	−0.0051 (8)	0.0003 (7)	0.0029 (8)
C5	0.0192 (9)	0.0286 (10)	0.0156 (9)	−0.0079 (8)	0.0043 (7)	−0.0029 (7)
C6	0.0154 (9)	0.0233 (10)	0.0202 (9)	−0.0046 (8)	0.0035 (7)	−0.0041 (7)
C7	0.0172 (9)	0.0175 (9)	0.0162 (8)	0.0000 (7)	0.0043 (7)	−0.0041 (7)
C8	0.0177 (9)	0.0232 (10)	0.0150 (8)	−0.0038 (7)	0.0023 (7)	−0.0002 (7)
C9	0.0190 (9)	0.0230 (10)	0.0144 (8)	−0.0041 (7)	0.0037 (7)	0.0013 (7)
C10	0.0171 (9)	0.0309 (11)	0.0230 (9)	0.0009 (8)	0.0044 (7)	0.0037 (8)
C11	0.0164 (9)	0.0389 (12)	0.0312 (11)	−0.0071 (9)	0.0044 (8)	0.0044 (9)
C12	0.0235 (10)	0.0276 (11)	0.0316 (11)	−0.0108 (8)	0.0035 (8)	0.0012 (9)
C13	0.0201 (9)	0.0207 (10)	0.0247 (10)	−0.0018 (8)	0.0055 (8)	−0.0008 (8)
C14	0.0177 (9)	0.0208 (10)	0.0211 (9)	−0.0025 (8)	0.0039 (7)	−0.0011 (8)
C15	0.0229 (10)	0.0295 (11)	0.0250 (9)	−0.0061 (8)	0.0090 (8)	0.0001 (8)
C16	0.0159 (9)	0.0172 (9)	0.0233 (9)	0.0016 (7)	0.0046 (7)	0.0018 (7)
C17	0.0182 (9)	0.0224 (10)	0.0214 (9)	0.0024 (8)	0.0031 (7)	0.0020 (7)

### Geometric parameters (Å, º)

**Table:**

S1—C2	1.7814 (16)	C9—C10	1.390 (2)
S1—C9	1.7831 (18)	C10—C11	1.385 (3)
N1—C7	1.291 (2)	C10—H10	0.9500
N1—C8	1.403 (2)	C11—C12	1.384 (3)
N2—C7	1.368 (2)	C11—H11	0.9500
N2—C14	1.470 (2)	C12—C13	1.383 (2)
N2—C16	1.472 (2)	C12—H12	0.9500
C1—C6	1.397 (2)	C13—H13	0.9500
C1—C2	1.398 (2)	C14—C15	1.526 (2)
C1—C7	1.502 (2)	C14—H14A	0.9900
C2—C3	1.395 (2)	C14—H14B	0.9900
C3—C4	1.388 (2)	C15—H15A	0.9800
C3—H3	0.9500	C15—H15B	0.9800
C4—C5	1.385 (2)	C15—H15C	0.9800
C4—H4	0.9500	C16—C17	1.521 (2)
C5—C6	1.383 (2)	C16—H16A	0.9900
C5—H5	0.9500	C16—H16B	0.9900
C6—H6	0.9500	C17—H17A	0.9800
C8—C13	1.403 (2)	C17—H17B	0.9800
C8—C9	1.406 (2)	C17—H17C	0.9800
			
C2—S1—C9	96.16 (8)	C9—C10—H10	119.9
C7—N1—C8	124.28 (14)	C12—C11—C10	119.53 (17)
C7—N2—C14	125.08 (14)	C12—C11—H11	120.2
C7—N2—C16	118.54 (14)	C10—C11—H11	120.2
C14—N2—C16	114.71 (13)	C13—C12—C11	120.46 (17)
C6—C1—C2	118.19 (15)	C13—C12—H12	119.8
C6—C1—C7	120.08 (15)	C11—C12—H12	119.8
C2—C1—C7	121.64 (14)	C12—C13—C8	121.32 (17)
C3—C2—C1	120.48 (15)	C12—C13—H13	119.3
C3—C2—S1	119.67 (13)	C8—C13—H13	119.3
C1—C2—S1	119.79 (13)	N2—C14—C15	112.82 (14)
C4—C3—C2	120.26 (16)	N2—C14—H14A	109.0
C4—C3—H3	119.9	C15—C14—H14A	109.0
C2—C3—H3	119.9	N2—C14—H14B	109.0
C5—C4—C3	119.52 (16)	C15—C14—H14B	109.0
C5—C4—H4	120.2	H14A—C14—H14B	107.8
C3—C4—H4	120.2	C14—C15—H15A	109.5
C6—C5—C4	120.27 (15)	C14—C15—H15B	109.5
C6—C5—H5	119.9	H15A—C15—H15B	109.5
C4—C5—H5	119.9	C14—C15—H15C	109.5
C5—C6—C1	121.19 (16)	H15A—C15—H15C	109.5
C5—C6—H6	119.4	H15B—C15—H15C	109.5
C1—C6—H6	119.4	N2—C16—C17	113.16 (14)
N1—C7—N2	118.19 (15)	N2—C16—H16A	108.9
N1—C7—C1	124.74 (14)	C17—C16—H16A	108.9
N2—C7—C1	116.77 (14)	N2—C16—H16B	108.9
N1—C8—C13	117.10 (16)	C17—C16—H16B	108.9
N1—C8—C9	125.17 (16)	H16A—C16—H16B	107.8
C13—C8—C9	117.30 (15)	C16—C17—H17A	109.5
C10—C9—C8	121.12 (16)	C16—C17—H17B	109.5
C10—C9—S1	119.41 (14)	H17A—C17—H17B	109.5
C8—C9—S1	119.46 (13)	C16—C17—H17C	109.5
C11—C10—C9	120.26 (17)	H17A—C17—H17C	109.5
C11—C10—H10	119.9	H17B—C17—H17C	109.5

### Hydrogen-bond geometry (Å, º)

Cg is the centroid of the C8–C12 ring.

**Table:**

*D*—H···*A*	*D*—H	H···*A*	*D*···*A*	*D*—H···*A*
C5—H5···N1^i^	0.95	2.70	3.576 (2)	154
C4—H4···*Cg*^i^	0.95	2.81	3.5759 (18)	139

Symmetry code: (i) *x*, −*y*+1/2, *z*+1/2.
